# Optical and magneto-optical properties of Gd_x_Fe_(100-x)_ thin films close to the compensation point

**DOI:** 10.1038/s41598-019-52252-z

**Published:** 2019-11-12

**Authors:** Eva Jesenská, Takayuki Ishibashi, Lukáš Beran, Martin Pavelka, Jaroslav Hamrle, Roman Antoš, Jakub Zázvorka, Martin Veis

**Affiliations:** 1 Department of Materials Science and Technology, Nagaoka University of Technology, Nagaoka, Niigata 1603-1 Japan; 2 Institute of Physics, Charles University in Prague, Prague, 12116 Czech Republic

**Keywords:** Magnetic properties and materials, Spintronics

## Abstract

Unlike ferromagnetic materials, ferrimagnetic metals have recently received considerable attention due to their bulk perpendicular magnetic anisotropy, low net magnetization and tunable magnetic properties. This makes them perfect candidates for the research of recently discovered spin-torque related phenomena. Among other ferrimagnetic metals, GdFe has an advantage in relatively large magnetic moments in both sublattices and tunability of compensation point above the room temperature by small changes in its composition. We present a systematic study of optical and magneto-optical properties of amorphous Gd_x_Fe_(100-x)_ thin films of various compositions (x = 18.3, 20.0, 24.7, 26.7) prepared by DC sputtering on thermally oxidized SiO_2_ substrates. A combination of spectroscopic ellipsometry and magneto-optical spectroscopy in the photon energy range from 1.5 to 5.5 eV with advanced theoretical models allowed us to deduce the spectral dependence of complete permittivity tensors across the compensation point. Such information is important for further optical detection of spin related phenomena driven by vicinity of compensation point in nanostructures containing GdFe.

## Introduction

Amorphous ferrimagnetic thin films composed of rare earth elements and transition metals attracted considerable attention because of their useful technological applications^1–4^. As one of their important representatives, Gd_x_Fe_(100-x)_ has significant advantages, such as large magnetization and possibility to adjust its compensation temperature, coercive field and saturation magnetization by changing the composition^5–7^. These properties make Gd_x_Fe_(100-x)_ substantial for modern micro- and nano-electronic research, where it is often used in domain wall junctions or magneto-optical (MO) memories^1,3,4^. Recently, a novel concept of high speed MO spatial light modulator for holographic displays based on GMR with Gd_x_Fe_(100-x)_ as a free layer was proposed^2^.

Another valuable feature of Gd_x_Fe_(100-x)_ is that it enables direct access to its spins through the electromagnetic interactions, which makes this material subject of importance for future magnetic recording (such as heat assisted magnetic recording) and information processing technologies. Recent numerical atomic scale modeling simulations of the spin dynamic in Heisenberg Gd_x_Fe_(100-x)_ ferrimagnet demonstrated that the rapid transfer of energy into the spin system leads to switching of the magnetization within a few ps without the necessity of applied magnetic field. The experiment in GdFeCo alloys, which used linearly polarized fs laser pulse to produce the ultra-fast heating, confirmed this prediction^8–10^. Moreover, by using circularly polarized laser pulses, it is possible to take an advantage of the magnetic circular dichroism effect to record a magnetic domain in which the helicity of the laser pulse influences the final magnetization direction^8,10–12^. These mechanisms allow the Gd_x_Fe_(100-x)_ magnetic domain light spin manipulation and hence coherent control of the magnetization precession at fluencies as low as 6 μJ/cm^2^ ^13^ and in rates of ps^10,11,14^.

The main purpose of our investigation was the determination of the complete permittivity tensors of Gd_x_Fe_(100-x)_ thin films with various compositions (x = 18.3, 20.0, 24.7, 26.7) since their spectra provide a deeper look at optical and MO properties of this material. Moreover, the knowledge of the complete tensor allows the theoretical prediction of complex physical properties in complicated multilayered nanostructures containing Gd_x_Fe_(100-x)_ layer.

In this work, we used spectroscopic ellipsometry (SE) at energies from 1.5 to 6 eV and MO spectral measurements at energies from 1.5 to 5.5 eV. From SE data, we derived the diagonal permittivity tensor elements ε_1r_ and ε_1i_ spectra of Gd_x_Fe_(100-x)_ thin films. We examined MO properties by polar MO Kerr effect (MOKE) rotation and ellipticity measurements. From these results we determined the spectral dependence of the off-diagonal Gd_x_Fe_(100-x)_ permittivity tensor elements ε_2r_ and ε_2i_. We also performed MOKE hysteresis loop measurements, which demonstrated changes in magnetization in dependence on Gd_x_Fe_(100-x)_ composition.

Gd_x_Fe_(100-x)_ is usually covered by a capping to avoid the oxidation process^15^. However, this fact complicates its analysis. Optical properties of capping materials (here Ru, SiO_2_) may slightly differ in dependence on material they are deposited on. The reason behind this behavior is usually the lattice mismatch between the film and substrate, which induces strains of various kinds^16–18^. In order to deal with this issue, we used two different capping materials which allowed more precise determination of Gd_x_Fe_(100-x)_ permittivity tensors. Measurement of SE showed very similar optical properties of individual Gd_x_Fe_(100-x)_ compositions for both cappings.

## Results and Discussion

**Figure 1:**
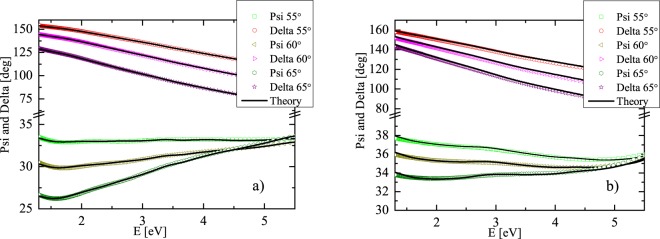
Measured variable angle SE Psi and Delta spectra of (**a**) Gd_20_Fe/Ru and (**b**) Gd_20_Fe/SiO_2_ samples compared to the theoretical model.

We parameterized spectral dependences of optical functions (diagonal elements of permittivity tensor) to ensure KK consistent results. We used the summation of two Lorentz oscillators and Drude term in the spectral range from 1.5 to 6 eV. The parameters of the dispersion functions were fitted together with thicknesses of individual layers of investigated samples. Figure 1 shows that this theoretical approach describes both Gd_20_Fe_80_/Ru and Gd_20_Fe_80_/SiO_2_ SE experimental spectra adequately. Sample parameters and optical properties of Si, SiO_2_ and Ru, used in the SE analysis are shown in the Methods section.

**Figure 2:**
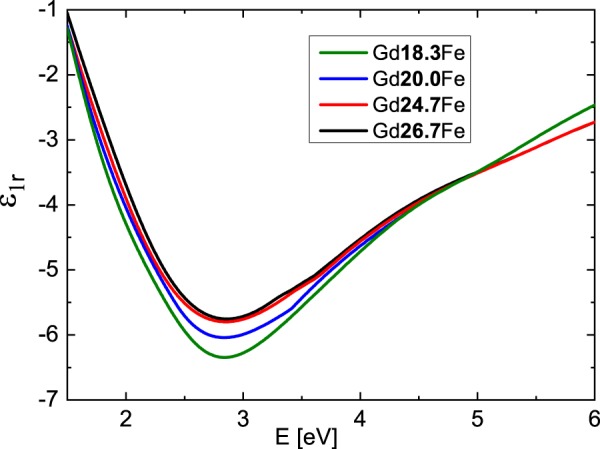
Real parts of diagonal elements of the permittivity tensor of the Gd_x_Fe_(100-x)_ thin films.

**Figure 3:**
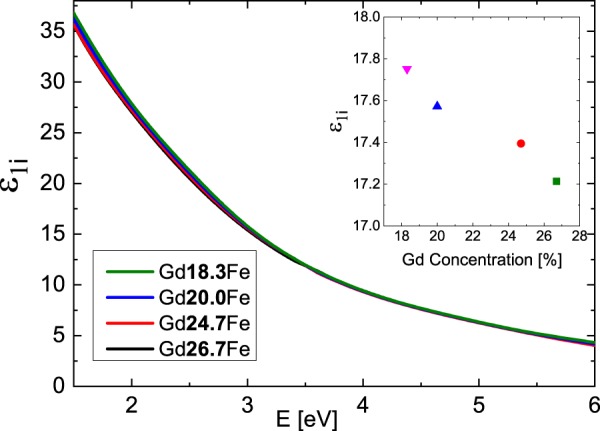
Imaginary parts of diagonal elements of the permittivity tensor of the Gd_x_Fe_(100-x)_ thin films. The inset displays the dependence of the imaginary parts on Gd concentration at E = 2.8 eV.

Figure 2 shows derived spectral dependences of the real part of the diagonal permittivity tensor elements ε_1r_ while Fig. 3 shows the spectra of the imaginary parts ε_1i_. The ε_1r_ spectra are dominated by one global minimum at 2.9 eV while the ε_1i_ amplitudes decrease at higher energies for all compositions. Obtained results show spectral behavior similar to previously published optical properties of Gd_22_Fe_78_^19^, Fe, Gd^20,21^ and Gd_x_Fe_(100-x)_^6,22^. The spectral dependence of ε_1r_ between 1.5 and 3 eV, where ε_1r_ decreases its amplitude with increasing energy, differs from typical Drude-like behavior (describing intra-band transitions) of metallic compounds, and is similar to the behavior of some transitions metals (including Cr, Gd, Ru, Ti^20,21^). This behavior is coming from the Lorentz contribution centered near 1.9 eV and most likely originates in the inter-band transition, which involves Fe 3d and Gd 5d states. The Fe 3d state lie around 1.5 eV below Fermi energy, while Gd 5d states are situated approx. 0.5 eV above the Fermi energy^23,24^. The second Lorentz oscillator centered near 2.5 eV does not significantly modify the spectral dependence of Drude behavior due to its small amplitude. This points on the origin of Gd d-d electron transition^23,24^, since this transition should be forbidden with small oscillator strength.

Finally, we discuss the Gd substitution effect. With increase of the Gd content the amplitude of the first Lorentz function around 1.9 eV decreased (see Table 2 in Methods). This is visible in Fig. 2 as a change of ε_1r_ amplitude around 2.9 eV and in the inset of Fig. 3. Increasing Gd content decreases Fe density of states below Fermi energy, resulting in the suppression of Fe 3d to Gd 5d transition probability. In contrast to that, the increase of Gd content is increasing the amplitude of the second Lorentz function centered near 2.5 eV. Such behavior supports the assignment of the origin to Gd d-d transition.

**Figure 4:**
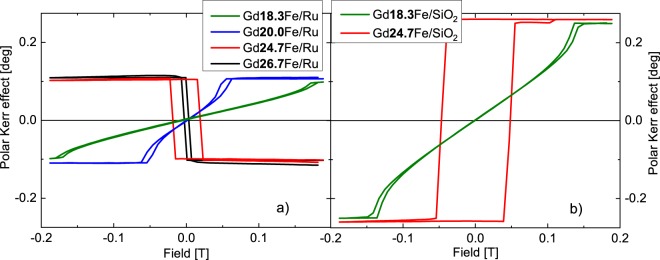
Hysteresis loops of examined samples with (**a**) Ru and (**b**) SiO_2_ cappings at 2.38 eV.

Polar MOKE hysteresis loop measurements are shown in Fig. 4. Samples with higher Gd content exhibit square-like loops with sharp transition, indicating out-of-plane easy axis of the net magnetization. As the composition approaches the compensation point, the coercivity increases. Below the composition value of x ≈ 25, the Fe moments become more dominant forcing the sample accommodate the in-plane anisotropy. The higher net magnetization value results in stronger effects of dipolar interaction, inducing a complex multi-domain state in the sample with negligible coercivity. With lower Gd content, the hysteresis loop shape becomes more prolonged and together with the increase of the saturation field hints on tilted direction of the net magnetization to the OOP orientation. Such state is described by complex butterfly-like shaped hysteresis loops shown in Fig. 4 for samples Gd_18.3_Fe and Gd_20_Fe.

Opposite direction of the magnetization as well as different coercivity for samples with the same Gd concentration x = 24.7, but different capping (Gd_24.7_Fe/Ru and Gd_24.7_Fe/SiO_2_) is noticeable in Fig. 4. Since this composition is extremely close to the compensation point, it would be reasonable to assume that the real composition of the sample might slight differ from the measured one (obtained integrally by EDX). Therefore, the sample with SiO_2_ capping might have lower concentration than x = 24.7 (situated below compensation point) and can be closer to the compensation point, since the coercivity is increasing when approaching the compensation. On the other hand, one cannot also exclude the influence of the capping layer itself, although its influence will be much smaller with respect to the layer/capping thickness ratio. Finally, the slight change in the temperature during the measurement can bring the sample above/below the compensation point.

**Figure 5:**
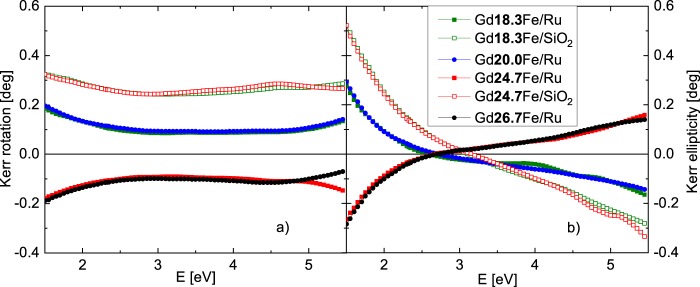
Polar MOKE (**a**) rotation and (**b**) ellipticity spectra of examined samples.

Figure 5 shows polar MOKE rotation and ellipticity spectra. Firstly, both spectra are characteristic by increasing value of rotation and ellipticity towards smaller energies. Secondly, samples with SiO_2_ capping show notably higher MO signal than samples with Ru capping (also visible in hysteresis loops measurements in Fig. 4). Because both capping materials have different optical properties the total reflection coefficient *r*_*pp*_ of the sample will differ (see Eq. 8 in Methods section). This influences the value of MOKE rotation, as well as ellipticity. Moreover, one can see that the substitution of Gd is slightly increasing amplitudes of the MOKE. Finally, as expected, amplitudes of MOKE rotation and ellipticity change their sign when Gd reaches the compensation concentration (x ≈ 25) and therefore when the net magnetization direction changes. All the data correspond to the hysteresis loops measurements.

**Figure 6:**
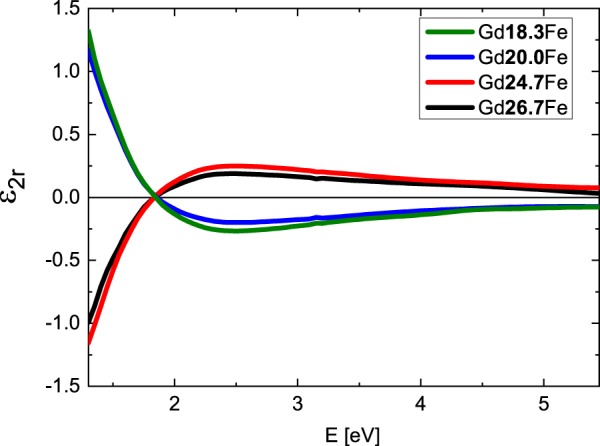
Real parts of the off-diagonal elements of the permittivity tensors of Gd_x_Fe_(100-x)_.

**Figure 7:**
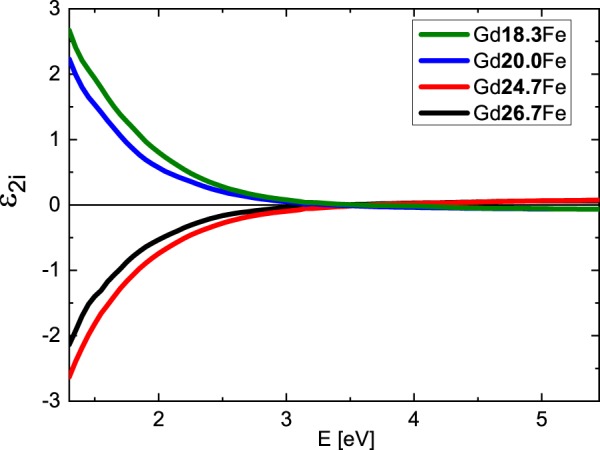
Imaginary parts of the off-diagonal elements of the permittivity tensors of Gd_x_Fe_(100-x)_.

Combining the results from SE and MOKE measurements we calculated the off-diagonal elements of the Gd_x_Fe_(100-x)_ permittivity tensors. In the calculation we used the thicknesses of the layers determined by SE. Figure 6 shows calculated spectrally dependent real parts of the off-diagonal elements ε_2r_ and Fig. 7 imaginary parts of the off-diagonal elements ε_2i_. The spectra are consistent with the spectral behavior of diagonal elements ε_1_ and agree with previously reported results on Gd_22_Fe_78_^19^. From Figs 6 and 7 one can see a change in the sign when crossing the compensation point. This confirms the change of the direction of the net magnetic moment in the investigated samples. Apart from the sign change, the spectral behavior remains the same for all samples (it is clearly demonstrated by the same zero crossing near 1.8 eV in the Fig. 6). Magneto-optical Kerr effect mainly originates from electric-dipole transitions between particular spin-orbit split bands, which leads to the dispersion of the off-diagonal elements ε_2r_. Since the zero crossing in the Fig. 6 is situated near 1.8 eV for all samples with different composition, the electronic transitions have similar energy in all samples. This indicates only small changes in electronic structure of Gd_x_Fe_(100-x)_ with composition around the compensation point. The slight change in the amplitude of the off-diagonal components of the permittivity tensor can be attributed to the change of the net magnetization near the compensation point. The results are consistent with hysteresis loop measurements in Fig. 4.

The combination of SE and MOKE spectroscopy allowed us to successfully determine the spectral dependence of full permittivity tensor of Gd_x_Fe_(100-x)_. The analysis of optical properties revealed contribution of intra- and inter-band transitions described by Drude and Lorentz approaches. The inter-band transitions were assigned to Fe-Gd and Gd-Gd 3d bands. MOKE hysteresis loops confirmed the changes in magnetic ordering when the composition crosses the compensation point. Complex shape of hysteresis loops suggests multidomain structure after crossing the compensation point. MOKE spectroscopy and subsequent calculation of off-diagonal permittivity elements demonstrated, in agreement with SE, only small changes in electronic structure in the vicinity of the compensation point.

## Methods

### Theory

It is a common approach to describe the response of a material to electromagnetic wave in the presence of external magnetic field by permittivity and permeability tensors. At optical frequencies, we can assume the permeability to be unit scalar^25^. Since the magnetic field acts as a small perturbation of the system, the permittivity tensor, as a function of magnetization, can be expanded in the series to the second order as

1 $${\varepsilon }_{ij}={\varepsilon }_{ij}+{K}_{ijk}{M}_{k}+{G}_{ijkl}{M}_{k}{M}_{l},$$

where *ε*_*ij*_ is the permittivity independent on magnetization, *M*_*k*_,*M*_*l*_ are the components of magnetization vector and *K*_*ijk*_ and *G*_*ijkl*_ are linear and quadratic magneto-optical tensors, respectively. In the present work, we restrict ourselves only to the linear magneto-optical effect and omit the third term in Eq. (1).

If the magnetization is parallel to the z-axis of the Cartesian coordinate system (the magnetic film-ambient interface is normal to the z-axis, light is propagating along the z-axis) and if we restrict ourselves to linear MO effects, the permittivity tensor simplifies to the form^26^

2 $$(\begin{array}{ccc}{\varepsilon }_{1} & -i\cdot {\varepsilon }_{2} & 0\\ i\cdot {\varepsilon }_{2} & {\varepsilon }_{1} & 0\\ 0 & 0 & {\varepsilon }_{1}\end{array}).$$

Generally, the tensor elements are complex having real and imaginary parts:

3 $$\begin{array}{ccc}{\varepsilon }_{1} & = & {\varepsilon }_{1r}-i\cdot {\varepsilon }_{1i}\\ {\varepsilon }_{2} & = & {\varepsilon }_{2r}-i\cdot {\varepsilon }_{2i}\end{array}.$$

In the base of *s* and *p* polarized incident light, we can describe the optical response of a sample upon light reflection within the framework of the Jones matrix of reflection^25^

4 $${J}_{sp}^{R}=(\begin{array}{cc}{r}_{ss} & {r}_{sp}\\ {r}_{ps} & {r}_{pp}\end{array}){J}_{sp}^{I},$$

where its elements are amplitude reflection coefficients.

It is possible to derive the diagonal elements of the permittivity tensor analyzing the experimental data from SE. The change in the polarization state of the reflected light can be expressed the by the SE parameters Psi (ψ) and Delta (Δ), which are defined as

5 $$\tan \,{\rm{\psi }}\cdot {e}^{i\Delta }=\rho =\frac{{r}_{pp}}{{r}_{ss}}.$$

In this equation, tan *ψ* is the magnitude of the reflectivity ratio and Δ is the phase change between *s* and *p* polarized light. The important part of SE analysis is the proper parametrization of the dispersion relations of investigated material. In this work, we used Kramers-Kronig (KK) consistent Lorentz and Drude approaches. The phenomenological Lorentz approach is commonly used to describe the dispersion of inter-band transitions in matter as

6 $${\varepsilon }_{{1}_{Lorentz}}=\frac{A\Gamma {E}_{0}}{{E}_{0}^{2}-{E}^{2}-i\cdot E\Gamma }.$$

Here, the parameters E_0_, A, Γ denote the transition energy, amplitude and the broadening parameter respectively^27^ (p. 343)^28^. The Drude approach was used to model the free carrier contribution to the optical properties using the following equation

7 $${\varepsilon }_{{1}_{Drude}}=\frac{-{\hslash }^{2}}{{\varepsilon }_{0}\rho (\tau {E}^{2}+i\hslash E)}.$$

Parameters ρ and τ denote the resistivity and mean scattering time respectively.

It is possible to derive the off-diagonal elements of the permittivity tensor from spectroscopic MOKE data analysis. Here, we used the Yeh matrix formalism^25,26,29^ for theoretical calculations in studied multilayers. This formalism solves the wave equation in optically anisotropic layers, finds the eigenstates of propagation and subsequently uses the boundary conditions to relate the propagation across all layers. As a result, the Jones reflection and transmission matrices, containing Fresnel reflection coefficients, are obtained. In the polar MOKE experiment, we can express the change in the polarization state of the reflected beam by the complex MO Kerr angle Φ_K_, which is for incident *p*-polarization and small angles defined as follows

8 $${\Phi }_{K}={\theta }_{K}-i\cdot {e}_{K}=\frac{{r}_{sp}}{{r}_{pp}}.$$

In this equation, ϴ_k_ is the Kerr rotation, e_k_ is the Kerr ellipticity. To derive the off-diagonal elements of permittivity tensor, we considered a model of semi-infinite Si substrate and three additional layers. Each layer was characterized by the complex permittivity tensor and the thickness. Combining the experimental values of MOKE, Eq. (8) and Yeh formalism, we were able to fit the off-diagonal elements ε_2_ for each energy of incident light.

### Samples and measurement details

Thin films of Gd_x_Fe_(100-x)_, *x* = [18.3–26.7], were deposited by DC sputtering on thermally oxidized silicon substrates (100) in Kr gas (8.7 × 10^−2^ Pa). The growth rate was 3.6 nm/min. Ru capping was grown by the same technique. SiO_2_ capping was deposited by ion beam sputtering technique with *rf* ion source. Table 1 shows the model structures and nominal thicknesses (determined by XRF) used for the theoretical analysis of SE and MOKE experimental data. The thicknesses of capping layers were chosen to be smaller compared to the penetration depth of the incident light in the whole spectral range. Model structures contained Si substrate, 300 nm thick layer of SiO_2_, 100 nm thick layer of Gd_x_Fe_(100-x)_, and a 3 nm thick capping Ru layer or 20 nm thick SiO_2_ layer on top. A surface roughness was modelled as a separate layer using a Bruggeman Effective Medium Approximation formula^30^ of the mixture of 50% of hosting material (ε) and 50% of void. The roughness was deduced as a thickness of this layer.

**Table 1:** Structure compositions and nominal thicknesses of examined samples.

	Substrate	Layer 1	Layer 2	Layer 3	Indication of samples in figures and text
Sample 1	Si	SiO_2_ (300 nm)	Gd**18**.**3**Fe**81**.**7** (100 nm)	Ru (3 nm)	Gd**18**.**3**Fe /Ru
Sample 2	Si	SiO_2_ (300 nm)	Gd**18**.**3**Fe**81**.**7** (100 nm)	SiO_2_ (20 nm)	Gd**18**.**3**Fe /SiO_2_
Sample 3	Si	SiO_2_ (300 nm)	Gd**20**.**0**Fe**80**.**0** (100 nm)	Ru (3 nm)	Gd**20**.**0**Fe /Ru
Sample 4	Si	SiO_2_ (300 nm)	Gd**24**.**7**Fe**75**.**3** (100 nm)	Ru (3 nm)	Gd**24**.**7**Fe /Ru
Sample 5	Si	SiO_2_ (300 nm)	Gd**24**.**7**Fe**75**.**3** (100 nm)	SiO_2_ (20 nm)	Gd**24**.**7**Fe /SiO_2_
Sample 6	Si	SiO_2_ (300 nm)	Gd**26**.**7**Fe**73**.**3** (100 nm)	Ru (3 nm)	Gd**26**.**7**Fe /Ru

A Mueller matrix ellipsometer Woollam RC2 was employed to perform SE measurements of ellipsometric parameters Psi and Delta in the spectral range from 1.5 to 6 eV for incident angles 55°, 60° and 65°. Obtained experimental data were analyzed using the CompleteEase software. In the case of samples with both cappings (Gd_18.3_Fe_81.7_, Gd_24.7_Fe_75.3_) a “Multi Sample Analysis” was used. This allowed to simultaneously fit the optical properties of GdFe from both samples together while keeping optical properties of Ru and SiO_2_ constant^27^.

The spectral dependences of optical functions (diagonal elements of permittivity tensor) were parametrized with the summation of two Lorentz oscillators and Drude term in the spectral range from 1.5 to 6 eV to ensure KK consistent results. The parameters of the dispersion functions were fitted together with thicknesses of individual layers of investigated samples. Used parameters are listed in Table 2. Optical properties of Si, SiO_2_ and Ru, used in the SE analysis, were determined from SE measurements on individual samples. Resulting thicknesses of individual layers of investigated samples are summarized in Table 3. To verify the thicknesses of Gd_x_Fe_(100-x)_ layers obtained from SE, XRF measurements were performed as well, giving similar results, as follows from Table 3.

**Table 2:** Fitted parameters of Lorentz oscillators and Drude term used to parameterize optical functions of Gd_x_Fe_(100-x)_ optical functions in the spectral range from 1.5 to 6 eV.

	Lorentz 1			Lorentz 2			Drude term		
	E (eV)	A	Γ (eV)	E (eV)	A	Γ (eV)	ρ (Ωm)	τ (fs)	ε_inf_
Gd**18**.**3**Fe**81**.**7**	1.89	6.66	2.30	2.56	1.10	1.09	0.0160	0.105	2.26
Gd**20**.**0**Fe**80**.**0**	1.88	6.23	2.43	2.54	1.28	1.43	0.0162	0.106	2.28
Gd**24**.**7**Fe**75**.**3**	1.84	6.04	2.70	2.49	1.67	1.84	0.0167	0.105	2.29
Gd**26**.**7**Fe**73**.**3**	1.84	5.82	2.83	2.41	1.67	1.99	0.0168	0.104	2.31

In here, E stands for central energies of oscillators; A represents amplitudes and Γ broadenings. For Drude model, ρ represents resistivity and τ mean scattering time.

**Table 3:** Fitted thicknesses used for the model of Gd_x_Fe_(100-x)_ in SE and X-ray in the spectral range from 1.5 to 6 eV.

	t_SiO2_ (nm) SE	t_GgFe_ (nm) SE	t_GgFe_ (nm) X-ray	t_Ru_ (nm) *capping* SE	t_SiO2_ (nm) *capping* SE	r (nm) SE
Gd**18**.**3**Fe**81**.**7**/**Ru**	307	131.6	136.9	3.1	—	2
Gd**18**.**3**Fe**81**.**7**/**SiO**_**2**_	307	130	136.9	—	11.3	0.3
Gd**20**.**0**Fe**80**.**0**/**Ru**	307	103	99.6	2.9	—	2
Gd**24**.**7**Fe**75**.**3**/**Ru**	307	95	87.7	2.7	—	1.9
Gd**24**.**7**Fe**75**.**3**/**SiO**_**2**_	307	87.7	87.7	—	10.5	0.7
Gd**26**.**7**Fe**73**.**3** /**Ru**	307	93.4	93.4	2.2	—	1.9

In here, t stands for thickness and r for roughness of the film interface with the ambient air.

Similarly to our previous work^19^, MOKE rotation and ellipticity spectra in the polar configuration were obtained using a method of generalized MO ellipsometry with rotating analyzer, which allowed the determination of the rotation angles with high accuracy. All the spectra were measured at the room temperature and nearly normal light incidence. Applied magnetic field was 1.2 T, which was enough for magnetic saturation of all the samples. Incident light was *p-*polarized. The data were recorded in the photon energy range from 1.5 to 5.5 eV.

Differential intensity detection method was employed to acquire MOKE rotation hysteresis loops at 2.38 eV. We performed all measurements in the polar geometry and at the room temperature. Applied magnetic field was ranging from −1.8 T up to 1.8 T, which was beyond saturation point.
